# An Extract of *Artemisia dracunculus* L. Promotes Psychological Resilience in a Mouse Model of Depression

**DOI:** 10.1155/2018/7418681

**Published:** 2018-05-13

**Authors:** Jun Wang, Adelaida Esteban Fernández, Simoni Tiano, Jing Huang, Elizabeth Floyd, Alexander Poulev, David Ribnicky, Giulio M. Pasinetti

**Affiliations:** ^1^Department of Neurology, Icahn School of Medicine at Mount Sinai, New York, NY 10029, USA; ^2^Geriatric Research, Education and Clinical Center, James J. Peters Veterans Affairs Medical Center, Bronx, NY 10468, USA; ^3^Pennington Biomedical Research Center, Baton Rouge, LA 70808, USA; ^4^Department of Plant Biology, Rutgers, The State University of New Jersey, New Brunswick, NJ 08901, USA

## Abstract

Stress-induced peripheral inflammation contributes to depression-like behaviors in both human and experimental models. PMI 5011, a botanical extract of *Artemisia dracunculus* L., was previously shown to have multiple bioactivities, including anti-inflammatory activity. In this work, using a repeated social defeat stress (RSDS) model of depression, we demonstrate that oral administration of the botanical extract PMI 5011 promotes resilience to RSDS-mediated depression-like phenotypes. We also show that the behavioral improvements are associated with attenuation of stress-mediated induction of inflammatory cytokines in the periphery and alteration of synaptic plasticity in the nucleus accumbens (NAc). Our studies provide experimental evidence that botanical extracts such as PMI 5011, which target pathological mechanisms (i.e., peripheral inflammation) not addressed by currently available antidepressants, could be further developed as novel therapeutics for the treatment of stress disorders and anxiety in humans.

## 1. Introduction

Depression and anxiety are widespread psychological conditions with broad health implications. Currently available antidepressant treatments are mainly designed to target the serotonergic and/or the noradrenergic system in the brain. Approximately half of the patients, however, do not fully respond to the approved antidepressants [1], and these treatments are often associated with therapeutic time lag and a wide range of undesirable “adverse” events [2]. This may reflect the heterogeneity of the mechanisms underlying depression, highlighting an urgent need for new therapeutic targets that are not addressed by standard antidepressants.

Depression is a multicausal disorder and the underlying etiology and pathophysiology are not completely understood. Peripheral inflammation has received increasing attention in the past two decades. Many neuroimmune factors have been implicated in depressive disorders. Clinical studies report higher levels of circulating proinflammatory cytokines, such as interleukin-1*β*, interleukin-6 (IL-6), and tumor necrosis factor-*α* (TNF-*α*), in patients with major depressive disorder (MDD) [3–5]. How peripheral inflammation may modulate depression phenotypes is currently under intense investigation. More recently, it was reported that in both human and rodent models of depression, chronic social stress alters blood vessel ultrastructure and, in combination with stress-induced peripheral inflammation, increases blood brain barrier (BBB) leakiness that allows the infiltration of inflammatory molecules into the brain [6]. These inflammatory molecules, once in the brain, can act directly on neurons or indirectly through modulation of microglia and/or other CNS immune cells leading to alteration of neuroplasticity and the development of depression-like behaviors [6, 7]. This is further supported by the observation that intracranial infusion of proinflammatory cytokine IL-6 increases depression-associated behavior [8], and systemic treatment with monoclonal IL-6 antibody can effectively reduce circulating IL-6 and promote resilience to chronic social stress-induced depression-like behaviors [9]. These studies suggest that modulations of peripheral inflammation and associated immune signaling pathways may provide novel therapeutic strategies to prevent and/or treat depression.

Therapeutic interventions derived from natural origin are receiving increased attention due to their lack of adverse secondary effects and multitargeting mechanisms of action which may increase the likelihood of therapeutic efficacy [10]. *Artemisia dracunculus* L. (Russian tarragon) is a culinary herb that has many healthy properties. Several bioactive constituents have been described in *Artemisia dracunculus*, including flavonoids (flavones, flavanones, dihydroflavanols, and chalcones) and phenolic acids (hydroxybenzoic, caffeic, or 5-*O*-caffeoylquinic acids, among others), as well as small amounts of sesquiterpenoids or vitamins. However, its composition widely varies depending on the plant phenotype and geographic origin [11]. Various bioactivities have been described in rodents, including strong anti-inflammatory, hepatoprotective, antihyperglycemic, antihyperlipidaemic, and antioxidant activities.

PMI-5011 is a well-characterized ethanol extract of *Artemisia dracunculus* L. that has been studied for at least 18 years and is the subject of many published research articles. Some of the earlier studies were focused on the identification of specific bioactive compounds using bioactivity-guided fractionation. The characterization of the extract was published over a series of research articles [12] and was summarized very succinctly in a review article by Schmidt et al. [13]. Three distinct assays were used independently to identify 6 bioactive compounds including 4,5-di-*O*-caffeoylquinic acid, davidigenin, 6-demethoxycapillarisin, 2′,4′-dihydroxy-4-methoxydihydrochalcone, 2′,4-dihydroxy-4′-methoxydihydrochalcone, and sakuranetin. The structures of these compounds were confirmed using LC-MS and 2D NMR, and their potential antidiabetic activities were tested both *in vitro* [14, 15] and *in vivo* in rodent models of type 2 diabetes [14, 16, 17]. For example, 2′,4′-dihydroxy-4-methoxydihydrochalcone is bioactive *in vitro* to inhibit (1) aldose reductase enzyme, (2) protein tyrosine phosphatase 1B activity and expression, and (3) phosphoenolpyruvate carboxykinase overexpression and its activities, validated *in vivo* by demonstrating acute glucose-lowering effects in mice [14]. The exact molecular mechanisms underlying the effect of PMI 5011 on diabetes are still under active investigation. However, *in vitro* studies have demonstrated that PMI 5011, at 10 *μ*g/ml, can significantly improve insulin and insulin receptor signaling in primary human skeletal muscle cells [16] whereas in cultured human primary skeletal muscle myoblasts, at 5 *μ*g/ml, PMI 5011 notably attenuates inflammatory response to cytokine stimuli through inhibition of nuclear factor-*κ*B (NF-*κ*B) signaling [17]. The anti-inflammatory activity of PMI 5011 was demonstrated in mouse and human pancreatic cells, whereas at 5-10 *μ*g/ml, it reduces nitric oxide (NO), NO synthase activity (iNOS), and IL-6 [18]. *In vivo* efficacies of PMI 5011 were also reported in several studies. For example, the administration of PMI 5011 (500 mg/kg/day) to mice fed on a high-fat diet showed antidiabetic effects comparable to conventional drug treatment, such as metformin (*P* < 0.05) [14]; the treatment with 500 mg/kg/day PMI 5011 for 7 weeks normalized glycemia (*P* < 0.01), alleviated nerve conduction slowing and sensory neuropathy (*P* < 0.05), and decreased lipoxygenase and nitrated protein accumulation in a mouse model of prediabetic neuropathy [19]. In a mouse model of diabetes, diet supplemented with 1% PMI 5011 for 8 weeks improved insulin signaling via Akt and IRS-associated PI3 kinase (*P* < 0.001) [20].

The extraction procedure for PMI 5011 from *Artemisia dracunculus* L. has been standardized and provides a consistent fingerprint of these bioactive compounds (Table 1).

Acute and chronic stress has long been used to model mood and anxiety disorders. In mice, a variety of physiological and psychological stressors have been shown to produce behaviors resembling depression-like symptoms [21–25], among which the repeated social defeat stress (RSDS) model recapitulates many key behavioral features associated with psychosocial stress in humans. The RSDS paradigm consists of repeated subordinations of an experimental C57BL/6 mouse by an aggressive dominant CD-1 mouse, leading to long-lasting behavioral consequences. As in humans, chronic social subordination of susceptible mice leads to a spectrum of depression-like behaviors, among which social avoidance and anhedonia are most relevant to human depression. Similar to human psychopathology, in which some individuals develop depression while others do not, a subset of resilient mice resist the development of such behaviors following RSDS [26]. Depression and anxiety are associated with functional abnormalities in brain regions involved in fear conditioning and emotion regulation [27], and these pathogenic alterations likely contribute to the vulnerability of certain individuals for developing depression/anxiety. Similarly, RSDS mice exhibit stress-induced abnormalities in synaptic remodeling, which include altered synaptic strength and connectivity [28–31] in the nucleus accumbens (NAc), a brain structure important for the development of anxiety/depression in response to trauma-related stimuli. Moreover, in the RSDS model, leukocyte-derived IL-6 regulates susceptibility versus resilience to stress, emphasizing the key role of peripheral IL-6 in depression [9, 32].

Based on the important contribution of peripheral inflammation in the pathophysiology of depression and the established anti-inflammatory activity of PMI 5011, we hypothesized that the administration of PMI 5011 may be able to attenuate depression-like phenotypes through modulation of stress-induced peripheral inflammation. In this study, we tested the preclinical efficacy of PMI 5011 in modulating depression-like behavior in the repeated social defeat stress (RSDS) mouse model of depression.

## 2. Materials and Methods

### 2.1. Materials

PMI 5011, a dried ethanolic extract of *Artemisia dracunculus* L., was prepared and analyzed as previously described [14]. Briefly, the harvested shoots were heated with 80% ethanol (*v*/*v*) to 80°C for 2 h. The extraction continued for an additional 10 h at 20°C. The extract was then filtered through cheese cloth and evaporated with a rotary evaporator. The aqueous extract was freeze-dried for 48 h, and the dried extract was homogenized with a mortar and pestle. The composition of bioactive components of PMI 5011 used in this study is shown in Table 1.

### 2.2. Animals

All C57BL/6J male mice were purchased from the Jackson Laboratory (Stock number 000664). Retired breeder CD-1 mice were purchased from Charles River Laboratory. All animals had access to regular chow ad lib and were maintained on a 12 : 12 h light/dark cycle with lights on at 07:00 h in a temperature-controlled (20 ± 2°C) vivarium, and all procedures were approved by the Institutional Animal Care and Use Committee (Protocol number IACUC-2014-0081).

### 2.3. Treatment

The male C57BL/6J mice (*n* = 22 per group) were group housed (*n* = 4-5 per cage) until the initiation of RSDS. The number of mice was calculated based on our previous social interaction studies employing RSDS. Power calculation found that 15 mice/group will have 90% statistical power to detect 25% (0.32 log2) fold change. Due to the nature of the defeat and associated injury, we used a larger number of mice (*n* = 22/group) to ensure that we will have sufficient statistical power to identify the behavioral changes in the event that not all mice complete the study. All mice were fed with a polyphenol-free diet for 10 days and were then randomly grouped into two groups: one group received a regular diet (OpenStandard Diet, D11112201, Research Diets) and the other group was treated with the same diet with 1% PMI 5011 incorporated (OpenStandard Diet, D17020901, Research Diets, Table 2), starting 2 weeks prior to RSDS and throughout RSDS and SI testing. The dose we use is a standard dose we used in all the preclinical studies conducted in diabetes research. The dose is well tolerated and has consistently produced improvement in glucose metabolism and inflammation [14, 20, 33–36]. Potential toxicity of PMI 5011 has been thoroughly tested, and we have established that dosage up to 1000 mg/kg/day for 90 days appears to be safe and nontoxic [37]. The treatment duration was based on our previous study demonstrating the efficacy of a bioactive dietary polyphenol-rich preparation in the same RSDS model [32]. One mouse from the vehicle-treated control group had to be euthanized due to the injury sustained from the RSDS. All remaining mice were subjected to SI. Twenty-four hours after the SI, one set of mice (*n* = 8 per group) was sacrificed for plasma cytokine and brain synaptic protein expression analysis without other behavioral testing, as splash testing and sucrose preference testing can potentially influence the reward circuit, which will affect synaptic gene expression. The other set of mice (*n* = 13 for the control group and *n* = 14 for the treatment group) was subjected to sucrose preference and splash testing.

### 2.4. Behavioral Testing

#### 2.4.1. RSDS

RSDS was performed as previously described [26, 38]. CD-1 mice were screened for aggressive characteristics prior to the start of social defeat experiments based on previously described criteria [26]. Specifically, CD-1 mice were individually caged, and on the day of screening, a C57BL/6J mouse was placed directly into the home cage of the CD-1 mouse for 180 seconds. The latency to aggression was noted and the same procedure repeated for two more times in the next two days, each time with a different C57BL/6J mouse as screener. The CD-1 mouse that (1) successfully attacked in at least two consecutive sessions and (2) has the latency to initial aggression less than 60 seconds was chosen and housed within the social defeat cage (26.7 w × 48.3 d × 15.2 h cm; Allentown Inc.) 24 hours prior to the start of defeats on one side of a clear, perforated plexiglass divider (0.6 × 45.7 × 15.2 cm; Nationwide Plastics). The RSDS was conducted every day under regular house light. Briefly, the mice subjected to RSDS were exposed to a novel CD-1 aggressor mouse for 10 minutes once per day, over 10 consecutive days. Following the 10 minutes of interaction, the experimental C57/BL6J mice were removed to the opposite side of the social defeat cage, and sensory contact during the following 24-hour period was allowed. The C57BL/6 mice were returned to a single house following the last defeat and before the social avoidance testing.

#### 2.4.2. Social Avoidance Test (Social Interaction Test)

Social interaction (SI) testing was performed as previously described [26]. All SI tests were performed under red light conditions. The mice were placed in a novel interaction open-field arena custom-crafted from opaque plexiglass (42 × 42 × 42 cm; Nationwide Plastics) with a small animal cage placed at one end. Their movements were then automatically monitored and recorded (Ethovision 3.0; Noldus Information Technology) for 2.5 minutes in the absence (target absent phase) of a novel CD-1 mouse. This phase is used to determine baseline exploratory behavior. We then immediately measured 2.5 minutes of exploratory behavior in the presence of a caged CD-1 mouse (target present phase), again recording the total distance travelled and the duration of time spent in the interaction and corner zones. SI behavior was then calculated as total time spent in each zone or as a ratio of the time spent in the interaction with the target present divided by the time spent in the interaction zone with the target absent. All mice with a ratio above 1.0 were classified as resilient whereas below 1.0 were classified as susceptible.

#### 2.4.3. Splash Test

Following the SI testing, a sucrose splash test was carried out in the home cage under a red light [39]. Briefly, the mice were sprayed with 200 *μ*l of a 10% (wt/vol) sucrose solution directly onto the animal's back using a small atomizer to induce grooming behavior. The grooming frequency and latency were recorded for 5 minutes and manually scored.

#### 2.4.4. Sucrose Preference Testing

Following the last defeat, the mice were habituated to 50 ml tubes with a sipper top (a two-bottle choice) filled with drinking water. After the splash testing, the mice were given access to a two-bottle choice of water or 1% sucrose solution, and the consumption of each solution was recorded once every 24 hours for 48 hours. Sucrose preference was calculated as a percentage of sucrose consumption over total liquid consumption.

### 2.5. RNA Isolation and Gene Expression Assessment

One set of mice (*n* = 8) was sacrificed by decapitation without anesthesia 24 hours following the SI test. Trunk blood was collected from each mouse in EDTA-coated tubes, and plasma was collected following centrifugation at 2000*g* for 15 minutes. Total RNA from brain NAc of each mouse was isolated using the RNeasy Mini Kit (Qiagen, Valencia, CA) and reverse transcribed. Gene expression was measured in 4 replicates by quantitative RT-PCR using Maxima SYBR Green Master Mix (Fermentas) in ABI Prism 7900HT. The following are primer sequences: mouse hypoxanthine phosphoribosyltransferase (HPRT) forward: CCCCAAAATGGTTAAGGTTGC, HPRT reverse: CCCCAAAATGGTTAAGGTTGC, Rac1 forward: GGTAGGTGATGGGAGTCAGC, Rac1 reverse: CTGAAGTGCGACACCACTGT, vGlut2 forward: GCTCACCTCTACCCTCAATATG, vGlut2 reverse: CCACTTGCTCCATATCCCATG, PSD95 forward: CGGGAGAAAATGGAGAAGGAC, PSD95 reverse: GCATTGGCTGAGACATCAAG, VGAT forward: ACGACAAACCCAAGATCACG, and VGAT reverse: AAGATGATGAGGAACAACCCC. HPRT expression level was used as an internal control. Data were normalized using the 2^-ΔΔCt^ method as previously described [40, 41]. Levels of target gene mRNAs were expressed relative to those in control mice and plotted in GraphPad Prism.

### 2.6. Plasma Collection and Multiplex ELISA Assay for Peripheral Cytokines

The plasma collected from the trunk blood (*n* = 8 per group, see above) was assayed for cytokine levels 24 hours after the SI test. Multiplex MAP mouse cytokine/chemokine panel (EMD Millipore) was used to measure the levels of 32 cytokines/chemokines following the manufacturer's instruction. Briefly, 12.5 *μ*l of plasma was incubated with the mouse cytokine/chemokine magnetic premixed beads at 4°C overnight and washed three times with the washing buffer, followed by incubation with mouse cytokine/chemokine detection antibodies for 1 hour at room temperature (RT). Streptavidin-phycoerythrin was then added and incubated for 30 minutes at RT followed by three times washing and subjected to analysis on Luminex 200® Instrument xPONENT3.1 (Luminex, Austin, TX).

### 2.7. Overall Statistics

All values are expressed as mean and standard error of the mean (SEM). Unpaired two-tailed Student's *t*-tests with Welch's correction were used. In all studies, outliers are defined as 2 standard deviations (SD) from the mean and were excluded. The null hypothesis was rejected at the 0.05 level. All statistical analyses were performed using Prism Stat program (GraphPad Software Inc.).

## 3. Results

### 3.1. Prophylactic Treatment with PMI 5011 Promotes Resilience to RSDS-Mediated Depression Phenotypes

**Figure 1:**
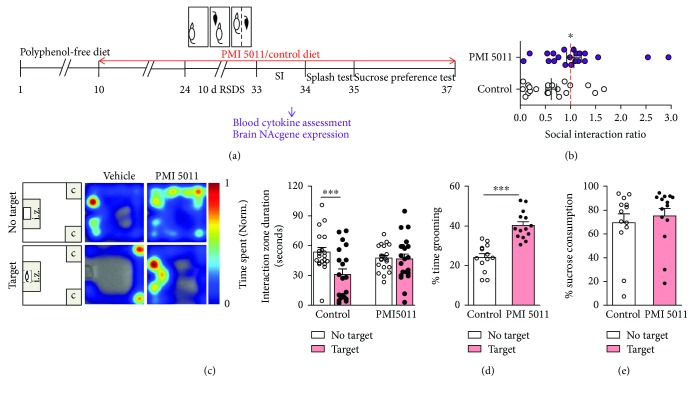
Oral administration of PMI 5011 promotes resilience to RSDS. (a) Schematic design of the experiment. (b) Treatment with PMI 5011 that increases the proportion of mice showing a resilient phenotype, as measured by the social interaction ratio (two-tailed unpaired *t*-test, *t*_39_ = 2.786, *P* = 0.018, *n* = 20, 21 mice; one mouse from each group was excluded as outlier). (c) Representative heat maps (left) and bar graph (right) of the social avoidance behavioral test of duration spent in interaction zone (seconds) in the absence or presence of social target in vehicle- and PMI 5011-treated mice (vehicle group: two-tailed paired *t*-test, *t*_19_ = 4.552, *P* = 0.0002; PMI 5011 group: two-tailed paired *t*-test, *t*_20_ = 0.166, *P* = 0.870). (d) Splash test (two-tailed unpaired *t*-test, *t*_25_ = 6.031, *P* < 0.0001, *n* = 13, 14 mice). (e) Sucrose preference test (two-tailed unpaired *t*-test, *t*_25_ = 0.584, *P* = 0.565, *n* = 13, 14 mice). All bar graphs represent mean ± SEM, ^∗^*P* < 0.05, ^∗∗∗^*P* < 0.001.

To test the efficacy of PMI 5011 in stress-mediated depression, we treated C57BL/6 male mice with PMI 5011 or vehicle delivered through their diet for 2 weeks prior to and throughout RSDS and then performed social avoidance/interaction (SI) testing (Figure 1(a)). We found that treatment with PMI 5011 greatly increased the proportion of mice resilient to stress compared to the vehicle-treated animals (Figure 1(b), *P* < 0.05). Overall, over 50% of mice receiving PMI 5011 showed a resilient behavioral phenotype, whereas ~20% were resilient in the vehicle control group. Moreover, we found that there was a significant reduction of duration of time spent in the interaction zone (i.z.) in the presence of an interactive mouse (target) in the vehicle-treated group compared to that in the absence of the interactive mouse following RSDS (Figure 1(c), *P* < 0.001), while there was no difference in the time spent in the i.z. in the absence of the interactive mouse in the PMI 5011 group (Figure 1(c)).

We next conducted the splash test, a measure of stress-induced decreased self-care that is only reversible by chronic standard antidepressant treatment [9]. We found that mice from the PMI 5011-treated group spent significantly increased time grooming following aerosol delivery of a 10% sucrose solution to the fur compared to the vehicle-treated group (Figure 1(d), *P* < 0.001), suggesting PMI 5011 treatment attenuates stress-induced self-neglect behavior. Following the splash test, we then conducted a sucrose preference test to evaluate the effect of PMI 5011 on stress-induced anhedonia behavior. We found that both groups had similar average sucrose consumption implicating PMI 5011 treatment does not attenuate stress-induced anhedonia phenotypes (Figure 1(e)).

### 3.2. Effect of PMI 5011 on Stress-Mediated Peripheral Inflammation

**Figure 2:**
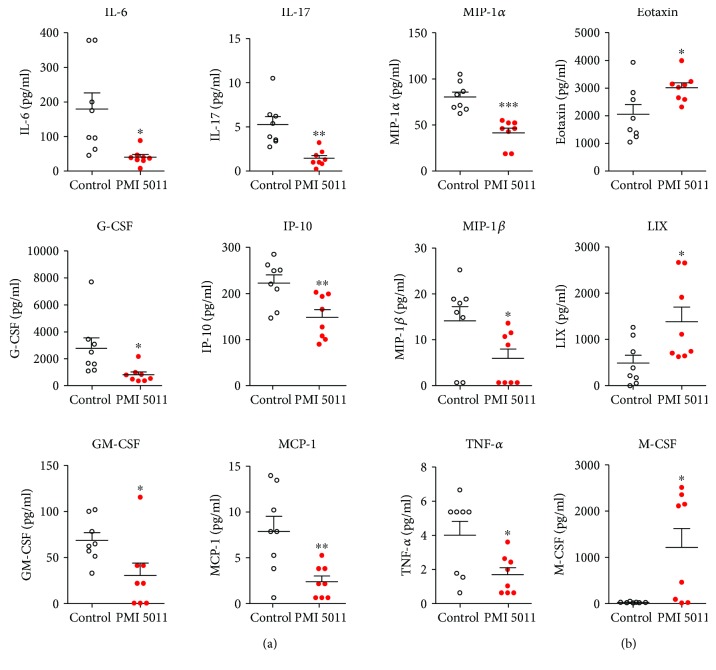
PMI 5011 treatment attenuates RSDS-induced inflammation in the periphery. (a) Plasma level of cytokines that was significantly reduced following PMI 5011 treatment 24 hours after last defeat (two-tailed unpaired *t*-test, *t*_14_ = 2.786, *P* = 0.0116 for IL-6; *t*_14_ = 4.019, *P* = 0.0013 for IL-17; *t*_14_ = 5.181, *P* = 0.0001 for MIP-1*α*; *t*_14_ = 2.469, *P* = 0.027 for G-CSF; *t*_14_ = 3.080, *P* = 0.0082 for IP-10; *t*_14_ = 2.196, *P* = 000455 for MIP-1*β*; *t*_14_ = 2.393, *P* = 0.0313 for GM-CSF; *t*_14_ = 3.146, *P* = 0.0072 for MCP-1 and *t*_14_ = 2.549, *P* = 0.0231 for TNF-*α*; *n* = 8 per group). (b) Plasma level of cytokines that was significantly increased following PMI 5011 treatment 24 hours after last defeat (two-tailed unpaired *t*-test, *t*_14_ = 2.429, *P* = 0.0292 for eotaxin; *t*_14_ = 2.487, *P* = 0.0261 for LIX; *t*_14_ = 2.706, *P* = 0.0180 for M-CSF, *n* = 8 per group). All bar graphs represent mean ± SEM, ^∗^*P* < 0.05, ^∗∗^*P* < 0.01, ^∗∗∗^*P* < 0.001.

As peripheral inflammation is our potential target for PMI 5011, we next measured the plasma level of cytokines 24 hours after the defeat. We found that, compared to the vehicle-treated group, PMI 5011-treated mice had significantly lower levels of IL-6, TNF-*α*, MCP-1, G-CSF, GM-CSF, IL-17, IP-10, MIP-1*α*, and MIP-*β* (Figure 2(a)). We also found that, compared to the vehicle-treated group, PMI 5011 treatment led to an increased level of eotaxin, LIX, and M-CSF (Figure 2(b)).

### 3.3. Effect of PMI 5011 on Stress-Mediated Synaptic Plasticity

**Figure 3:**
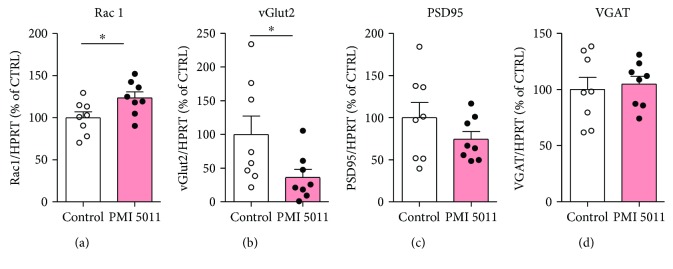
PMI 5011 treatment normalizes the expression of stress-induced excitatory synaptic protein in the NAc (a) Expression of Rac1 in the NAc (*t*_14_ = 2.354, *P* = 0.0337, *n* = 8 per group). (b, c) Expression of glutamatergic neuron markers vGlut2 and PSD95 (*t*_14_ = 2.137, *P* = 0.050 for vGlut2; *t*_14_ = 1.265, *P* = 0.2264 for PSD95, *n* = 8 per group). (d) Expression of GABAergic neuron marker VGAT (*t*_14_ = 0.3679, *P* = 0.7184, *n* = 8 per group). All bar graphs represent mean ± SEM, ^∗^*P* < 0.05.

We previously found that, in both humans and rodents, chronic stress reduces the expression of RAS-related C3 botulinum toxin substrate 1 (*Rac1*) in the NAc and stress-mediated downregulation of Rac1 in the NAc correlates with social avoidance behavior in the RSDS model of depression [28]. We showed that downregulation of Rac1 is necessary and sufficient for social avoidance behavior and that pharmacological modulation of Rac1 attenuated stress-induced depression phenotypes [28, 32]. Moreover, Rac1 can also influence excitatory synapses, such as postsynaptic density protein 95 (PSD95) and vesicular glutamate transporter 2 (vGlut2) both *in vivo* and *in vitro* [28, 32]. We also demonstrated that peripheral inflammation can causally influence the expression of genes that are important for synaptic function in the NAc [32]. Therefore, we next measured the expression of synaptic protein in the NAc of mice following RSDS by real-time PCR. We found that there was an ~25% significant increase in the expression of Rac1 in the NAc in the PMI 5011-treated group compared to vehicle-treated mice (Figure 3(a), *P* < 0.050). Moreover, we found that treatment with PMI 5011 led to a significant reduction of >60% in the expression of vGlut2 (*P* < 0.050) and an ~25% reduction of PSD-95, however, did not reach statistical significance. Both vGlut 2 and PSD95 are markers of excitatory neurons that are shown to be increased in the NAc following RSDS. Consistent with our previous findings, there were no differences in the expression of GABAergic vesicular GABA transporter (VGAT) (Figures 3(b)–3(d)).

## 4. Discussion

Major depressive disorder is a psychiatric disease that is the fourth most common cause of disability worldwide. Molecular mechanisms underlying the pathophysiology of major depressive disorders (MDD) are very complex and are affected by genetic, environmental, and biological processes. Currently, three major aspects of depression are being actively investigated. First of all, depression is influenced by an imbalance of neurotransmitters and receptors, including serotonin, adrenaline, dopamine, and glutamate [42]. Secondly, depression is associated with the hyperactivity of immune inflammatory responses as manifested by elevated expression of proinflammatory molecules, such as IL-6 and TNF-*α*. The overall elevated status of inflammation together with neurovasculature pathology and impaired BBB structural function leads to malfunction of the brain circuits related to mood and anxiety [5]. Lastly, stress-induced depression causes a disruption in the normal synaptic plasticity and induces changes in brain architecture [43]. Conventional antidepressant therapies mainly target neurotransmitters and are associated with low overall treatment efficacy and various unwanted side effects. Therefore, therapy targeting inflammation and brain synaptic plasticity may provide novel treatment strategies for MDD.

In recent years, natural products, especially polyphenols, have received growing interest due to their potential benefits in treating psychiatric disorders. It is believed that their strong antioxidant and anti-inflammatory activities and their ability to modulate synaptic plasticity may contribute to their mechanisms of action [10, 42, 44]. PMI 5011 is an ethanol extract from *A. drancunculus*, characterized by a high content of secondary metabolites, including coumarins, flavonoids, and phenylpropanoid acids [11]. In this study, we demonstrated that treatment with PMI 5011 significantly attenuated stress-induced social avoidance and self-neglect behaviors in a mouse model of depression. Moreover, we found that the improvement of behavior was associated with significant reduction of inflammatory cytokines in the blood.

Previous studies demonstrated that increased glutamatergic transmission on ventral striatum medium spiny neurons (MSNs) mediates stress-induced susceptibility following RSDS [45, 46]. More recently, we showed a cause-effect relationship among leukocyte-derived proinflammatory responses, brain reward circuitry synaptic remodeling, and the manifestation of depression-like behavioral phenotypes [32]. Here, we demonstrated that PMI 5011 treatment also reduced the expression of excitatory markers in the NAc, which may contribute to the phenotypes we observed. This modulation of glutamatergic synapses could be a result of PMI 5011-mediated downregulation of peripheral inflammation. It is also possible that selected metabolites derived from PMI 5011 may pass the BBB and reach the brain to directly modulate synaptic plasticity.

We demonstrated that treatment with PMI 5011 protects against susceptibility to stress-mediated depression phenotypes by reducing peripheral inflammation and preserving synaptic plasticity in the NAc. Our observation is consistent with clinical and preclinical evidence that overly active peripheral inflammation processes involving inflammatory cytokines and disruptions in the normal synaptic plasticity responses in the NAc are two key pathological mechanisms underlying depression and anxiety. The efficacy of PMI 5011 in alleviating depression-like symptoms may also be suitable for treating other neuropsychological disorders such as posttraumatic stress disorder, traumatic brain injury-induced mood disorder, and bipolar depression, which share similar symptoms with MDD. Our evidence supports the development of PMI 5011 as a novel therapeutic agent to treat patients with treatment-resistant MDD, particularly among the majority of patients who are characterized as having high plasma levels of inflammatory cytokines [9]. Given the excellent safety profile of PMI 5011 [11] and its noted anti-inflammatory potential [17], it can be readily tested in clinical studies for the treatment of stress disorders and depression either alone or in combination with currently available antidepressants.

## Figures and Tables

**Table 1:** Relative concentrations of the bioactive compounds from PMI 5011. The relative concentrations of davidigenin, 6-demethoxycapillarisin, DMC-1, and sakuranetin were quantified based on the equivalents of DMC-2 as measured by LC-MS. The standard for DMC-2 was commercially synthesized. The fingerprint of PMI-5011 is consistently comprised of these compounds in approximately these ratios.

Bioactive components of PMI 5011	% of PMI-5011 extract (*w*/*w*)
Davidigenin	1.2%
6-Demethoxycapillarisin	0.74%
2′,4-Dihydroxy-4′-methoxydihydrochalcone (DMC-1)	0.63%
2′,4′-Dihydroxy-4-methoxydihydrochalcone (DMC-2)	2.5%
Sakuranetin	2.8%

**Table 2:** Diet formulation used in the study.

Product number	D11112201		D17020901	
	*OpenStandard Diet*		*OpenStandard Diet*	
	gm%	kcal%	gm%	kcal%
Protein	19	*20*	19	20
Carbohydrate	63	65	62	65
Fat	7	*15*	6	15
Total		*100*		*100*
kcal/gm	3.81		3.77	

*Ingredient*	gm	kcal	gm	kcal
Casein	200	*800*	200	*800*
L-Cystine	3	*12*	3	*12*
Cornstarch	381	*1524*	381	*1524*
Maltodextrin 10	110	*440*	110	*440*
Dextrose	150	*600*	150	*600*
Cellulose, BW200	75	*0*	75	*0*
Inulin	25	*37.5*	25	*37.5*
Soybean oil	70	*630*	70	*630*
Mineral mix S10026	10	*0*	10	*0*
Dicalcium phosphate	13	*0*	13	*0*
Calcium carbonate	5.5	*0*	5.5	*0*
Potassium citrate, 1 H2O	16.5	*0*	16.5	*0*
Vitamin mix V10001	10	*40*	10	*40*
Choline bitartrate	2	*0*	2	*0*
**PMI 5011**	**0**	**0**	**10.9**	**0**
Yellow dye number 5, FD&C	0.025	*0*	*0*	*0*
Red dye number 40, FD&C	0	*0*	0.025	*0*
Blue dye number 1, FD&C	0.025	*0*	0.025	*0*
*Total*	**1071.05**	**4084**	**1082**	**4084**
**PMI 5011 (%)**	**0**	**0**	**1.0074**	**0**

## Data Availability

The data used to support the findings of this study are available from the corresponding author upon request.

## References

[1] Krishnan V., Nestler E. J. (2008). The molecular neurobiology of depression.

[2] Ferguson J. M. (2001). SSRI antidepressant medications: adverse effects and tolerability.

[3] Dowlati Y., Herrmann N., Swardfager W. (2010). A meta-analysis of cytokines in major depression.

[4] Maes M. (1995). Evidence for an immune response in major depression: a review and hypothesis.

[5] Miller A. H., Raison C. L. (2016). The role of inflammation in depression: from evolutionary imperative to modern treatment target.

[6] Merad M., Manz M. G., Karsunky H. (2002). Langerhans cells renew in the skin throughout life under steady-state conditions.

[7] Banks W. A., Kastin A. J., Gutierrez E. G. (1994). Penetration of interleukin-6 across the murine blood-brain barrier.

[8] Meagher M. W., Johnson R. R., Young E. E. (2007). Interleukin-6 as a mechanism for the adverse effects of social stress on acute Theiler’s virus infection.

[9] Hodes G. E., Pfau M. L., Leboeuf M. (2014). Individual differences in the peripheral immune system promote resilience versus susceptibility to social stress.

[10] Sureda A., Tejada S. (2015). Polyphenols and depression: from chemistry to medicine.

[11] Obolskiy D., Pischel I., Feistel B., Glotov N., Heinrich M. (2011). *Artemisia dracunculus* L. (tarragon): a critical review of its traditional use, chemical composition, pharmacology, and safety.

[12] Logendra S., Ribnicky D. M., Yang H. (2006). Bioassay-guided isolation of aldose reductase inhibitors from *Artemisia dracunculus*.

[13] Schmidt B., Ribnicky D. M., Poulev A., Logendra S., Cefalu W. T., Raskin I. (2008). A natural history of botanical therapeutics.

[14] Ribnicky D. M., Kuhn P., Poulev A. (2009). Improved absorption and bioactivity of active compounds from an anti-diabetic extract of *Artemisia dracunculus* L.

[15] Govorko D., Logendra S., Wang Y. (2007). Polyphenolic compounds from *Artemisia dracunculus L.* inhibit PEPCK gene expression and gluconeogenesis in an H4IIE hepatoma cell line.

[16] Kheterpal I., Scherp P., Kelley L. (2014). Bioactives from *Artemisia dracunculus* L. enhance insulin sensitivity via modulation of skeletal muscle protein phosphorylation.

[17] Vandanmagsar B., Haynie K. R., Wicks S. E. (2014). *Artemisia dracunculus* L. extract ameliorates insulin sensitivity by attenuating inflammatory signalling in human skeletal muscle culture.

[18] Aggarwal S., Shailendra G., Ribnicky D. M., Burk D., Karki N., Qingxia Wang M. S. (2015). An extract of *Artemisia dracunculus* L. stimulates insulin secretion from *β* cells, activates AMPK and suppresses inflammation.

[19] Watcho P., Stavniichuk R., Ribnicky D. M., Raskin I., Obrosova I. G. (2010). High-fat diet-induced neuropathy of prediabetes and obesity: effect of PMI-5011, an ethanolic extract of *Artemisia dracunculus L.*.

[20] Wang Z. Q., Ribnicky D., Zhang X. H. (2011). An extract of *Artemisia dracunculus* L. enhances insulin receptor signaling and modulates gene expression in skeletal muscle in KK-A^y^ mice.

[21] Hunter R. G., McCarthy K. J., Milne T. A., Pfaff D. W., McEwen B. S. (2009). Regulation of hippocampal H3 histone methylation by acute and chronic stress.

[22] Golub Y., Mauch C. P., Dahlhoff M., Wotjak C. T. (2009). Consequences of extinction training on associative and non-associative fear in a mouse model of posttraumatic stress disorder (PTSD).

[23] Siegmund A., Dahlhoff M., Habersetzer U. (2009). Maternal inexperience as a risk factor of innate fear and PTSD-like symptoms in mice.

[24] Brinks V., de Kloet E. R., Oitzl M. S. (2009). Corticosterone facilitates extinction of fear memory in BALB/c mice but strengthens cue related fear in C57BL/6 mice.

[25] Hammamieh R., Chakraborty N., de Lima T. C. M. (2012). Murine model of repeated exposures to conspecific trained aggressors simulates features of post-traumatic stress disorder.

[26] Golden S. A., Covington H. E., Berton O., Russo S. J. (2011). A standardized protocol for repeated social defeat stress in mice.

[27] Shin L. M., Handwerger K. (2009). Is posttraumatic stress disorder a stress-induced fear circuitry disorder?.

[28] Golden S. A., Christoffel D. J., Heshmati M. (2013). Epigenetic regulation of RAC1 induces synaptic remodeling in stress disorders and depression.

[29] Christoffel D. J., Golden S. A., Heshmati M. (2012). Effects of inhibitor of *κ*B kinase activity in the nucleus accumbens on emotional behavior.

[30] Christoffel D. J., Golden S. A., Russo S. J. (2011). Structural and synaptic plasticity in stress-related disorders.

[31] Christoffel D. J., Golden S. A., Dumitriu D. (2011). I*κ*B kinase regulates social defeat stress-induced synaptic and behavioral plasticity.

[32] Wang J., Hodes G. E., Zhang H. (2018). Epigenetic modulation of inflammation and synaptic plasticity promotes resilience against stress in mice.

[33] Wicks S., Taylor C. M., Luo M. (2014). *Artemisia* supplementation differentially affects the mucosal and luminal ileal microbiota of diet-induced obese mice.

[34] Kirk-Ballard H., Wang Z. Q., Acharya P. (2013). An extract of *Artemisia dracunculus* L. inhibits ubiquitin-proteasome activity and preserves skeletal muscle mass in a murine model of diabetes.

[35] Ribnicky D. M., Roopchand D. E., Poulev A. (2014). *Artemisia dracunculus* L. polyphenols complexed to soy protein show enhanced bioavailability and hypoglycemic activity in C57BL/6 mice.

[36] Yu Y., Mendoza T. M., Ribnicky D. M. (2018). An extract of Russian tarragon prevents obesity-related ectopic lipid accumulation.

[37] Ribnicky D. M., Poulev A., O'Neal J. (2004). Toxicological evaluation of the ethanolic extract of *Artemisia dracunculus* L. for use as a dietary supplement and in functional foods.

[38] Krishnan V., Han M. H., Graham D. L. (2007). Molecular adaptations underlying susceptibility and resistance to social defeat in brain reward regions.

[39] Yalcin I., Aksu F., Belzung C. (2005). Effects of desipramine and tramadol in a chronic mild stress model in mice are altered by yohimbine but not by pindolol.

[40] Livak K. J., Schmittgen T. D. (2001). Analysis of relative gene expression data using real-time quantitative PCR and the 2^−ΔΔ*C*^T method.

[41] Wang J., Gong B., Zhao W. (2014). Epigenetic mechanisms linking diabetes and synaptic impairments.

[42] Maletic V., Robinson M., Oakes T., Iyengar S., Ball S. G., Russell J. (2007). Neurobiology of depression: an integrated view of key findings.

[43] Duman R. S., Aghajanian G. K., Sanacora G., Krystal J. H. (2016). Synaptic plasticity and depression: new insights from stress and rapid-acting antidepressants.

[44] Trebaticka J., Durackova Z. (2015). Psychiatric disorders and polyphenols: can they be helpful in therapy?.

[45] Francis T. C., Chandra R., Friend D. M. (2015). Nucleus accumbens medium spiny neuron subtypes mediate depression-related outcomes to social defeat stress.

[46] Christoffel D. J., Golden S. A., Walsh J. J. (2015). Excitatory transmission at thalamo-striatal synapses mediates susceptibility to social stress.

